# Comparison of Three Airway Maneuvers of Jaw Thrust, Two-Handed E-C Technique With Head in Neutral Position, and Two-Handed E-C Technique With Head Fully Extended: A Prospective, Randomized, Double-Blind Crossover Study

**DOI:** 10.7759/cureus.53791

**Published:** 2024-02-07

**Authors:** Karthikeyan Ramakkannu, Annu Theagrajan, Manjunath Prabhu, Venkateswaran Ramkumar

**Affiliations:** 1 Department of Anesthesiology, Devaki Speciality Hospital, Madurai, IND; 2 Anesthesiology, Sree Balaji Medical College and Hospital, Chennai, IND; 3 Department of Anesthesiology, Kasturba Medical College, Manipal, IND

**Keywords:** trauma resuscitation, oxygenation, 2-handed e-c technique, mask ventilation, jaw thrust, airway opening maneuvers

## Abstract

Background

Bag-mask ventilation is an essential life-saving skill. The E-C technique of mask holding is the most popular. In patients with suspected cervical injury, the jaw thrust maneuver is recommended instead of the E-C technique with head tilt-chin lift. Should jaw thrust fail to produce adequate chest rise, the operator is advised to switch to the E-C technique with the head tilt-chin lift maneuver with head extension as it is vital to move oxygen into the lungs. We hypothesized that the E-C clamp with the head in the neutral position without head tilt might permit adequate ventilation without producing excessive movement of the cervical spine, which in turn might translate as less strain to the cervical spine.

Methods

In this prospective, randomized, double-blind, crossover study, we evaluated the relative efficacy of three airway maneuvers in opening the airway in anesthetized and paralyzed adults: jaw thrust, two-handed E-C technique with head in the neutral position, and two-handed E-C technique with head fully extended. The tidal volume generated during mechanical ventilation using these three techniques was considered as the primary outcome. Seventy-two subjects were recruited for this trial and all three techniques of mask holding were performed in each of these subjects in a sequence as dictated by a randomization table.

Results

The jaw thrust technique provided a mean tidal volume significantly higher than the two-handed E-C technique, with the head in the neutral position (p<0.001). Similarly, the two-handed E-C technique with the head fully extended provided a mean tidal volume significantly higher than the two-handed E-C technique with the head in neutral position (p<0.011). The mean tidal volume obtained with jaw thrust and two-handed E-C technique with head fully extended were comparable (p=0.78).

Conclusion

The two-handed E-C technique with the head fully extended, and the jaw thrust technique both produce good and comparable tidal volumes. The two-handed E-C technique with the head in a neutral position provides adequate though lower tidal volumes as compared to the other two techniques.

## Introduction

Bag-mask ventilation is an essential, life-saving skill performed by the anesthesiologist. It enables ventilation and oxygenation of the patient till a definitive airway is secured [1,2]. Failure to establish a patent airway can lead to hypoxic brain injury. The E-C technique with head tilt-chin lift is the most popular technique for bag-mask ventilation [3]. Bag-mask ventilation may be provided by either a single person or two persons, where one person holds the mask while the other squeezes the bag. The one-handed technique is not effective at times, especially in obese or edentulous patients, leading to inadequate ventilation. In these situations, two-handed mask-holding can be adopted as an alternative technique [4-6]. In patients with suspected cervical spine injury, the E-C technique with head tilt-chin lift is not recommended; a jaw thrust maneuver is preferred [5]. Should jaw thrust fail to produce adequate chest rise, the operator is advised to switch to the E-C technique with head tilt-chin lift and extension of the head, as it is vital to move oxygen into the lungs [7].

We hypothesized that the E-C clamp technique with the head in a neutral position might permit adequate ventilation without producing excessive movement of the cervical spine, which in turn might translate as less strain to the cervical spine. The present study was conducted to assess the relative efficacy of three airway maneuvers in opening the airway in anesthetized and paralyzed adults: jaw thrust, two-handed E-C technique with head in the neutral position, and two-handed E-C technique with head fully extended. The tidal volume generated was taken as the marker of efficacy. We also evaluated the presence of a leak around the mask and epigastric leak.

## Materials and methods

The study was prospective, randomized, double-blind, and crossover in nature. Institutional Ethics Committee clearance from Kasturba Medical College, Manipal, was obtainedfor the study period of two years, and the study was registered with the Clinical Trial Registry of India (CTRI/2018/04/013069). Written informed consent was obtained from those who agreed to participate in the study. We evaluated the relative efficacy of three airway maneuvers in opening the airway in anesthetized and paralyzed adults: jaw thrust, two-handed E-C technique with head in the neutral position, and two-handed E-C technique with head fully extended. Taking a difference in tidal volume of 50 mL as clinically significant with a standard deviation of 135 mL as obtained in our pilot study, we determined a sample size of 72 patients with a power of 80% at a 95% confidence level.

**Figure 1:**
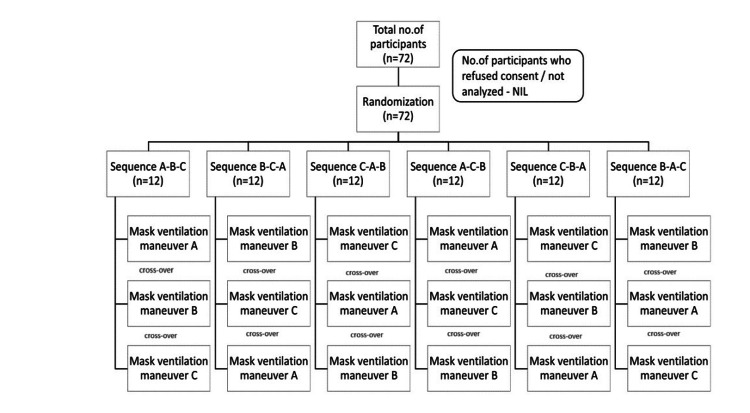
CONSORT diagram All participants were assigned to one of the six sequences using a random number table. The three maneuvers (A, B and C) of mask ventilation were done in all patients, starting with one maneuver and subsequently crossing over to the other two maneuvers as per the random number table. The sequences of mask holding were ABC, BCA, CAB, ACB, CBA and BAC. A: jaw thrust maneuver; B: two-handed E-C technique with head in a neutral position; C: two-handed E-C technique with head fully extended CONSORT - Consolidated Standards of Reporting Trials

Adults of either gender aged between 18 and 65 years belonging to the American Society of Anesthesiologists (ASA) with physical status 1 or 2 scheduled for elective surgical procedures under general anesthesia with muscle relaxation were included in the study. Those with a body mass index >30 kg/m², gastroesophageal reflux disease, predicted difficult mask ventilation (presence of beard, edentulous status, features of obstructive sleep apnea), or restriction of neck extension were excluded from the study. Seventy-two participants were studied, and none were lost to follow-up, as shown in Figure 1.

Observer 1 evaluated patients preoperatively, verified the inclusion criteria, explained the procedure to the patient, and obtained written informed consent. Observer 2, who was unaware of the mask-holding technique that was being used at that point of the study, recorded the tidal volume (primary objective) and the presence of an epigastric leak (secondary objective). Observer 3 mask-ventilated the patients, starting with the two-handed E-C technique with the head fully extended following anesthetic induction. 

All participants were assigned to one of six sequences, as indicated below by using a random number table. The sequence of mask holding was ABC, BCA, CAB, ACB, CBA and BAC. Patients were positioned on the operating table with no pillow under the head. The operating table height was adjusted such that it was at the level of the umbilicus of the operator who was performing the techniques (observer 3). The table height was not altered during the period of study when all three techniques of mask holding were being evaluated.

**Figure 2:**
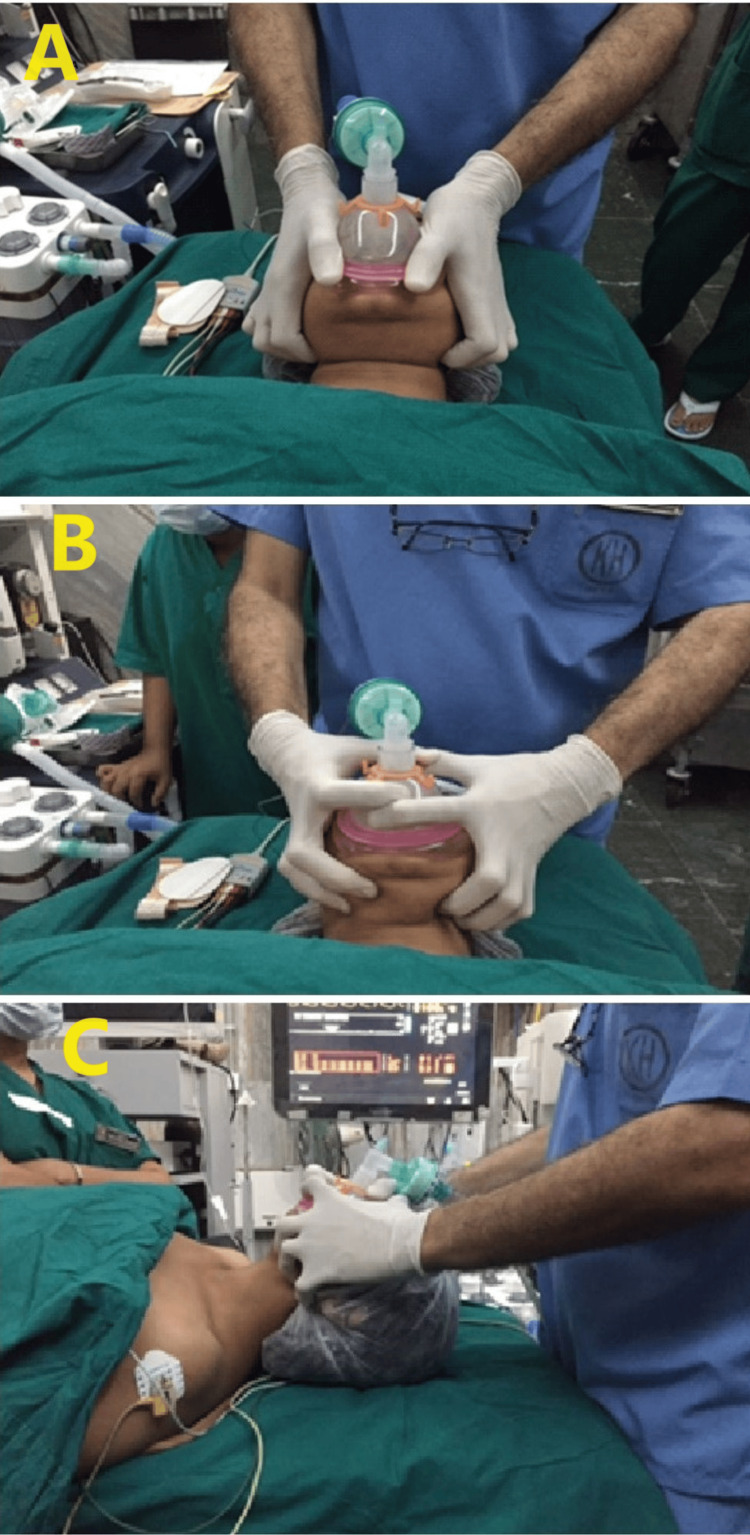
Three airway maneuvers A: jaw thrust maneuver; B: two-handed E-C technique with head in a neutral position; C: two-handed E-C technique with head fully extended

While performing a jaw thrust (A), the face mask was applied using a conventional jaw thrust maneuver with both hands to open the airway. The index finger, middle, ring, and little fingers placed along the horizontal ramus of the mandible provided the mandibular lift. The thumbs and thenar eminences placed along the sides of the mask were pushed down to achieve a good seal, as shown in Figure 2A.

While performing the two-handed E-C technique with the head in neutral position (B), the mask was applied using the E-C technique where the little, ring and middle fingers of both hands we­­­­re placed along either side of the body of the mandible forming an "E" while the index finger and thumb of both hands formed a semicircle or "C" around the mask to obtai­n a leak-proof fit of the mask over the face[6] (Figure 2B).

While performing the two-handed E-C technique with the head fully extended (C), the mask was applied using the E-C technique where the little, ring and middle fingers of both hands were placed along either side of the body of the mandible, forming an "E", while the index finger and thumb of both hands formed a semicircle or "C" around the mask to create a leak-proof fit of the mask over the face (Figure 2C).

The Spacelabs Anesthesia Workstation with Blease 900 series ventilator (ascending bellows) was used in this study. Following anesthetic induction and complete muscle paralysis as determined by the absence of a train-of-four response, the subjects were ventilated using a pressure control mode of ventilation with a peak inspiratory pressure (PIP) of 15 cm H_2_O, inspiration to expiration ratio (I:E) of 1:2, respiratory frequency of 10 breaths per minute (bpm) and a fresh gas flow rate of oxygen of 5 L/min. It was ensured that the ascending bellows would touch the top of the ventilator casing at the end of each expiration. Patients were randomly allocated to one of the sequences according to the random number table.

Induction was done with propofol 2 to 3 mg/kg, fentanyl 2 mcg/kg, and muscle paralysis was achieved with vecuronium 0.1 mg/kg. Patients were mask-ventilated using sevoflurane or isoflurane in oxygen till the absence of a train-of-four was observed. Bolus doses of intravenous mephentermine were administered if the mean arterial pressure was less than 60 mm Hg.

A consultant anesthesiologist (observer 3) performed the three techniques of mask holding as per the random table. To ensure adequate blinding, a screen was placed at the level of the nipples of the patient. Observer 3 could visualize chest rise, monitor the capnograph tracing on the patient monitor, and also note the presence of any audible mask leak (secondary objective). The anesthesia workstation was stationed behind observer 3 and hence, observer 3 was blinded to the tidal volume generated (primary outcome). Observers 2 and 3 were standing with their backs to each other, with observer 2 focusing attention on the workstation and recording the breath-by-breath tidal volumes being generated. Observer 2 also walked along a fixed path behind the workstation and the screen to auscultate for air leaks in the epigastric region for three consecutive breaths.

As mentioned above, observer 2 noted the tidal volume displayed on the ventilator screen and also auscultated the epigastric leak. The tidal volume display was observed over one minute for each technique. The tidal volume generated for each of the 10 breaths was noted, and an average value was calculated. Observer 3, who was in charge of providing bag-mask ventilation, was allowed to adjust the finger position for each technique during the initial two to three breaths, after which he was not allowed to change hand position until the measurements were completed. As this was a crossover design, following the completion of observations using one technique, the alternative techniques were performed as per the randomization table, and the parameters were recorded again. No airway adjuncts were used. When signs of inadequate mask ventilation were observed, such as tidal volume <150 mL, end-tidal carbon dioxide <10 mm Hg, or a SpO_2_ drop to <95%, the technique was considered unsuccessful, and an alternative technique was employed.

Statistical analysis to estimate the difference between the three techniques was performed using a two-way ANOVA test. The two-way ANOVA test compared the mean differences between groups that have been split into two independent variables. Data were analyzed using SPSS version 15.0 (SPSS Inc., Chicago, US). A p-value <0.05 was considered significant.

## Results

Seventy-two adults were included in the study, out of which 57 were female and 15 were males (see Table 1). While 47 patients had a body mass index (BMI) ranging from 18.4 to 24.9 kg/m^2^, the remaining 25 patients had a BMI ranging between 25 and 29.9 kg/m^2^.

**Table 1:** Demographic data of patients studied (n=72)

Demographic characteristic	Value
Gender (male/female)	15/57
Age (range, (years)	18-62
Height (range, cm)	141-181
Weight (range, kg)	42-80
Body mass index (range, kg/m^2^)	18.4-29.9

The mean tidal volume obtained with the jaw thrust technique, two-handed E-C technique with head in the neutral position, and two-handed E-C technique with head fully extended were 533±132 mL, 471±154 mL, and 521±142 mL, respectively (Table 2).

**Table 2:** Measured outcome parameters (n=72) * two patients had leaks with all three techniques Only one patient had both a mask leak and an epigastric leak.

Parameter	Jaw thrust	Two-handed E-C with head neutral	Two-handed E-C with head fully extended
Tidal volume (mLM; mean±SD)	533±132	471±154	521±142
Mask leak* (number of patients)	5	10	3
Epigastric leak (number of patients)	3	2	0

The jaw thrust technique provided a mean tidal volume of 533±132 mL, which was significantly higher than the two-handed E-C technique with the head in a neutral position (471±154 mL). The difference was statistically significant (p<0.001). Similarly, the two-handed E-C technique with the head fully extended provided a mean tidal volume of 521±142 mL, which was significantly higher than the two-handed E-C technique with the head in a neutral position (471±154 mL). This difference was also statistically significant (p=0.011; see Table 3).

**Table 3:** Intergroup comparison of tidal volumes * two-way ANOVA test was statistically significant

Technique 1	Technique 2	Mean difference in tidal volume (mL) between techniques 1 and 2	Standard error	p-value*
Jaw thrust	Two-handed E-C with head in neutral position	62.15	11.200	0.001*
Two-handed E-C with head fully extended	Two-handed E-C with head in neutral position	50.63	11.200	0.011*
Jaw thrust	Two-handed E-C with head fully extended	11.53	11.200	0.78

The mean tidal volumes obtained with jaw thrust and the two-handed E-C technique with the head fully extended were comparable (p=0.78; see Table 3). Even among overweight individuals with a BMI ranging from 25 to 29.9 kg/m^2^, the same trend continued. The incidence of mask leak was maximum with two-handed E-C technique with the head in neutral position (10/72), followed by jaw thrust (5/72) and two-handed E-C technique with the head fully extended (3/72). Two of the patients had mask leaks with all three techniques. Even though the epigastric leak was present in very few cases (5/72), it was observed with the jaw thrust maneuver (in two patients) and the two-handed E-C technique with the head in a neutral position (in three patients). No epigastric leak was noted during the two-handed E-C technique with the head fully extended.

## Discussion

The single-handed E-C technique is a widely used technique for providing bag-mask ventilation. If the single-handed E-C technique fails to provide adequate ventilation, two-handed mask holding employing either the E-C technique or jaw thrust technique may be adopted. When a two-handed technique is adopted to mask-ventilate the patient, a second person may be needed to provide ventilation by squeezing the bag. Alternately, the patient can be mechanically ventilated, as we did in our study. 

The most important cause of death following trauma is airway obstruction [8]. Airway maneuvers such as jaw thrust or head tilt-chin lift and bag-mask ventilation are provided to create a gas exchange. These maneuvers, however, are associated with cervical spine displacement and could have the potential to cause secondary neurological injury. The jaw thrust maneuver produces the least movement in unstable C1-C2 injury as compared to head tilt-chin lift and is, hence, the recommended technique in cases of cervical spine injury [9,10]. The efficacy of mask ventilation by the E-C technique without head extension in comparison with other techniques has not been studied previously.

Only a few studies have compared the efficacy of two-handed mask ventilation techniques. Joffe et al. studied the tidal volume obtained with the two-handed jaw thrust technique and single-handed E-C technique in 42 nonparalyzed adults. The tidal volume obtained with the two-handed jaw thrust technique was significantly greater (8.60±2.31 mL/kg) than the single-handed E-C technique (6.80±3.1 mL/kg). The authors included participants with the presence of predictors of difficult mask ventilation such as facial hair, BMI>30 kg/m^2^, edentulous status, history of snoring, and restriction of neck extension [6]. Hart et al. studied a simulated difficult airway model where the one-handed E-C technique yielded a median of 428.4 mL, the two-handed E-C technique yielded a median of 550.8 mL, and the two-handed V-E technique yielded a median of 538 mL. The authors concluded that there was no significant difference between the two-handed E-C technique and the two-handed V-E technique and that two-handed mask ventilation was more effective than the single-handed technique [11]. Our study was different as we studied the relative efficacy of jaw thrust (which is essentially two-handed), two-handed EC technique with head in the neutral position, and two-handed EC technique with head fully extended in anesthetized and paralyzed adults. We found that with patients being mechanically ventilated on pressure-controlled ventilation, both jaw thrust and two-handed E-C technique with head fully extended provided greater and comparable tidal volumes than the two-handed E-C technique with head in neutral position. However, our study did not include patients who were predicted to be difficult to mask-ventilate or intubate.

Even though the epigastric leak was present in very few instances (5/72), it was present only during the use of the jaw thrust maneuver (two participants) and the two-handed E-C technique with the head in neutral position (three participants). No epigastric leak was noted during the two-handed E-C technique with the head fully extended. The two-handed E-C technique with the head in a neutral position produced a mask leak in 10 out of 72 patients, which was the highest among the three techniques. Our study showed that even though the two-handed E-C technique with the head in a neutral position was the least efficient among the three techniques, this, too, consistently provided adequate tidal volume. This information can be used by personnel involved in trauma care. It would be appropriate to try the two-handed E-C technique with the head in the neutral position in case of an emergency with a suspected cervical spine injury before attempting to extend the neck if difficulty with jaw thrust is encountered. The use of an oropharyngeal airway can further improve ventilation and thereby decrease the mask leak. Essentially, this would indirectly be equivalent to a simulated scenario where manual-in-line stabilization is used during mask ventilation. Mask ventilation with neck extension could be considered the last option in such patients. In our study, no airway adjuncts such as nasopharyngeal airway and oropharyngeal airway were used in any patient as all three techniques provided tidal volumes >150 mL.

Our study had a few limitations. We studied patients scheduled for elective surgery with no predictors of difficult airway. Our patients were also paralyzed. Hence, our results may not apply to patients with predictors of difficult mask ventilation in emergencies and when muscle relaxation is inadequate. We mask-ventilated patients using mechanical ventilation with standardized ventilatory parameters. However, in true trauma emergencies, manual bag-mask ventilation will have to be employed, and in such situations, there may be heterogeneity in the PIP and tidal volume with each breath delivered [12]. Our understanding is that even a minimal PIP of 15 cm H_2_0 using manual bag-mask ventilation and either of the three techniques (including the two-handed E-C technique with the head in a neutral position) will prove to be adequate and life-saving. As per the protocol, only participants between the age group of 18 and 65 years were enrolled in the study, thereby excluding the pediatric and geriatric population in whom the incidence of both anticipated and unanticipated difficult airways is higher. Hence, the results of our study cannot be extrapolated to these patient subgroups. Around 79% of the patients included in our study were females. The jaw thrust technique provided the best tidal volume in this subgroup. In male patients who constituted the remaining, the two-handed E-C technique with the head fully extended provided the best tidal volume. Hence, the overall result of jaw thrust providing maximum tidal volume may not hold good for male patients. Although observer 3 was blinded to the tidal volume generated, being an experienced anesthesiologist, observer 3 could visualize the chest rise, monitor the capnograph tracing on the patient's monitor, and gauge the minute ventilation clinically, which is a practical limitation in our study. We propose that future studies should focus on patients with predictors of difficult mask ventilation and could include the use of an oral airway while evaluating the tidal volumes generated.

## Conclusions

Three techniques of mask ventilation were studied in anesthetized and paralyzed adult patients with no predicted difficulty in face mask ventilation or intubation. The two-handed E-C technique with the head fully extended produced a tidal volume of 521±142 mL, while the jaw thrust technique produced a tidal volume of 533±132 mL. Both these techniques produce good and comparable tidal volumes. The two-handed E-C technique with the head in the neutral position provided a tidal volume of 471±154 mL and was the least efficient of the techniques studied in terms of tidal volumes achieved. However, as even the lower tidal volumes provided by this technique are clinically adequate, the two-handed E-C technique with the head in the neutral position may be considered as an alternative to jaw thrust in patients with suspected cervical trauma.
